# Mr. Lewcock's Letter and the Hospital Saturday Executive

**Published:** 1913-02-22

**Authors:** A. W. Davis

**Affiliations:** Secretary. Hospital Saturday Fund, 54 Gray's Inn Road, W.C.


### The Editor's Letter-Box.

### Mr. Lewcock's Letter and the Hospital Saturday Executive.

*To the Editor of* The Hospital.

Sir,—The attention of the Executive Committee was drawn last night to a letter signed by Mr. G. A. Lewcock containing statements and opinions relating to the work of this Fund which appeared in your issue of the 8th inst. The Committee instructed me to inform you and the readers of The Hospital that this communication was not authorised by them, nor can they be responsible for the statements contained therein.—Yours faithfully,

A. W. Davis, Secretary.

Hospital Saturday Fund, 54 Gray's Inn Road, W.C., February 12, 1913.

### The Metropolitan Hospital Sunday Fund Meeting.

*To the Editor of* The Hospital.

